# Energy-Adjusted Dietary Inflammatory Index and Diabetes Risk in Postmenopausal Hispanic Women

**DOI:** 10.1016/j.jand.2023.08.002

**Published:** 2023-08-05

**Authors:** Monica D. Zuercher, Danielle J. Harvey, Lauren E. Au, Aladdin H. Shadyab, Margarita Santiago-Torres, Simin Liu, Nitin Shivappa, James R. Hébert, John A. Robbins, Lorena Garcia

**Affiliations:** University of California Davis, Davis, CA.; University of California Davis, Davis, CA.; University of California Davis, Davis, CA.; Herbert Wertheim School of Public Health and Human Longevity Science, University of California San Diego, La Jolla, CA.; Division of Public Health Sciences, Fred Hutchinson Cancer Research Center, Seattle, WA.; Public Health and Medicine Brown University, Providence, RI.; Cancer Prevention and Control Program & Department of Epidemiology and Biostatistics, Arnold School of Public Health, University of South Carolina, & Department of Nutrition, Connecting Health Innovations, LLC, Columbia, SC.; director, Cancer Prevention and Control Program & Department of Epidemiology and Biostatistics, Arnold School of Public Health, University of South Carolina, & Department of Nutrition, Connecting Health Innovations, LLC, Columbia, SC.; University of California Davis, Davis, CA.; University of California Davis, Davis, CA.

**Keywords:** Inflammatory diet, Diabetes, Hispanic or Latinos

## Abstract

**Background:**

Type 2 diabetes is a major public health concern in the United States and worldwide. The dietary inflammatory index (DII) and the energy-adjusted DII (E-DII) are tools that assess dietary inflammation. Previous evidence suggests that obesity can modify the association between inflammation and disease.

**Objective:**

The aim of this study was to evaluate the association between the DII/E-DII and incident diabetes in self-identified Hispanic women from the Women’s Health Initiative (WHI). The secondary aim was to evaluate whether obesity modifies the association between the DII/E-DII scores and incident diabetes.

**Design:**

Participants were from the WHI Observational Study and the Clinical Trial Components (except women from the treatment arm in the Dietary Modification Trial) conducted among postmenopausal women in the United States. DII/E-DII scores were calculated from a self-administered food frequency questionnaire at baseline that included 122 food items, of which 12 are representative of Hispanic eating patterns.

**Participants/settings:**

Participants included 3,849 postmenopausal women who self-identified as Hispanic that were recruited for the WHI from 1993 to 1998 at 40 US clinical centers.

**Main outcome measures:**

The outcome was incident diabetes.

**Statistical analysis performed:**

Cox regression models were used to assess the association between DII/E-DII and incident diabetes. Models were adjusted for age at baseline, lifestyle-related risk factors, known type 2 diabetes mellitus (T2DM) risk factors, and neighborhood socioeconomic status. Interaction was tested between the DII/E-DII scores and obesity.

**Results:**

The incidence of diabetes was 13.1% after a median follow-up of 13 years. Higher E-DII scores were associated with a higher risk of incident diabetes (hazard ratio [HR], 1.09; 95% confidence interval [CI], 1.04–1.14). There was no interaction between E-DII scores and obesity (*P* = 0.73).

**Conclusions:**

Pro-inflammatory diets, as measured by higher E-DII scores, were associated with a higher risk of incident diabetes. Future research is needed for understanding how the inflammatory potential of diets can be decreased.

THE INCREASE IN TYPE 2 DIABETES MELLITUS (T2DM) over the last decades represents a major public health concern because it is a leading cause of blindness, kidney failure, heart attack, stroke, and lower limb amputation.^1,2^ Moreover, health disparities in diabetes prevalence, complications, and comorbidities exist worldwide.^3^ In the United States, the prevalence of diabetes in Hispanic adults is 15.5% in comparison with 13.6% in non-Hispanic White adults.^4^ Among Hispanic heritage groups, adults with a Mexican origin have the highest prevalence (14.4%), followed by adults with a Puerto Rican origin (12.4%), Central/South American origin (8.3%), and Cuban origin (6.5%).^5^

Hispanic families residing in the United States experiencing food insecurity (15.8%) lack access to nutritious foods, placing them at risk of having a poor-quality diet, becoming overweight, and developing obesity.^6^ Furthermore, United States–born or second-generation Hispanic individuals tend to consume more unhealthy foods and have a greater energy intake than foreign-born Hispanic individuals, partially because of dietary acculturation.^7,8^

Chronic inflammation plays a key role in the pathogenesis of T2DM by increasing insulin resistance, *β*-cell dysfunction, and apoptosis.^9,10^ Pro-inflammatory effects have been attributed to the intake of various nutrients and foods, including intakes of saturated fatty acids, trans fatty acids, refined carbohydrates, ultraprocessed food, including processed meats, sugar-sweetened beverages, dairy products, and refined grains.^11-13^ In contrast, anti-inflammatory effects have been attributed to the intake of specific nutrients and foods, including intakes of dietary fiber, polyunsaturated fatty acids, fish, fruits, vegetables, and whole grains.^11-13^ The dietary inflammatory index (DII) is a scoring algorithm that assesses the overall inflammatory potential of an individual’s diet and has been validated to predict the concentration of inflammatory biomarkers.^14,15^ The energy-adjusted dietary inflammatory index (E-DII) is a modified version of the DII that was created to account for the relationship between energy and nutrient intakes.^16^ Therefore, it provides a convenient tool to assess the effect of dietary inflammation on the risk of diseases that have chronic inflammation as part of their pathophysiological processes.

Only one cross-sectional study has examined the association between DII and T2DM in Hispanic Populations.^17^ The study conducted by Denova-Gutiérrez et al^17^ in 1,174 adults from Mexico City found that participants with higher DII scores had higher odds of T2DM than participants with lower DII scores (odds ratio [OR]_Q5 vs. Q1_ = 3.02, *P* = 0.005).^17^ Three more studies have examined the association between the DII and risk factors for T2DM (ie, insulin resistance, fasting glucose levels, postload glucose levels, and prediabetes) in populations from Iran, South Africa, and the Netherlands, and results showed that higher DII scores were associated with increases in the risk factors for T2DM.^11,12,18^ The studies evaluating the association between the DII and T2DM are scarce and subject to methodological limitations, such as those encountered in cross-sectional or case-control designs, including potential information biases resulting in reverse causality.^19,20^ Therefore, the association between the DII and T2DM in Hispanic populations from diverse heritage backgrounds in the United States, who are at greater risk of T2DM than non-Hispanic populations, remains unclear.^16^

Furthermore, other factors such as obesity can alter the observed association between diet-associated inflammation and some health outcomes.^18,21^ The association between the DII and cardiovascular disease and interleukin-6 is stronger or only significant in participants with overweight or obesity but not in participants with a healthy weight.^22,23^ These findings suggest that pro-inflammatory diets may have a more detrimental effect on health when obesity is present, so more studies are needed to determine the association of obesity and its complex interaction with the inflammatory potential of the diet and health.

To fill the gap in evidence regarding the link between the DII/E-DII and T2DM in Hispanic populations and the role that obesity plays in this process, the current study evaluated the association between the DII/E-DII and incident diabetes among postmenopausal Hispanic women from the Women’s Health Initiative. The hypothesis was that higher DII/E-DII scores would be associated with a higher risk of incident diabetes.

## METHODS

### Participants

The Women’s Health Initiative (WHI) enrolled 161,808 postmenopausal women across 40 WHI clinical centers nationwide between October 1, 1993 and December 31, 1998. Participants in the WHI study ranged in age from 50 to 79 years at enrollment and were either randomized into one or more of three clinical trials (Hormone Therapy Trial, the Diet Modification Trial, and the Calcium and Vitamin D Trial) or enrolled into the observational study.^24^ The current study included 6,484 postmenopausal women who self-identified as Hispanic on a baseline demographic questionnaire (Chose “Hispanic/Latino [ancestry is Mexican, Cuban, Puerto Rican, Central American, or South American]” to answer the question *How would you describe your racial or ethnic group? If you are of mixed blood, which group do you identify with most?*). Participants with a history of diabetes at baseline (N = 610), assignment to the treatment arm in the diet modification Trial (N = 683), an implausible reported energy intake (<600 kcal/day or >5,000 kcal/day) (N = 347), use of nonsteroidal anti-inflammatory drugs (N = 882) or anti-diabetes drugs (N = 32), and missing information regarding incidence of diabetes during follow-up (N = 81) were excluded. After applying the exclusion criteria, the sample size for the current analyses was 3,849 postmenopausal Hispanic women (2,365 from the observational study and 1,484 from the clinical trials).

The WHI project was reviewed and approved by the Fred Hutchinson Cancer Research Center (Fred Hutch) Institutional Review Board in accordance with the US Department of Health and Human Services regulations at 45 CFR 46 (approval number: IR# 3467-EXT). Participants provided written informed consent to participate. Additional consent to review medical records was obtained through signed written consent. Fred Hutch has an approved Federal Wide Assurance on file with the Office for Human Research Protections under assurance number 0001920. The WHI is registered in ClinicalTrials.gov with the ID NCT00000611.

### Incident Diabetes

Incident diabetes was defined as a self-report of a new physician’s diagnosis of diabetes treated with hypoglycemic medication during follow-up.^25^ At each semi-annual or annual contact, participants were asked, “Since the date given on the front of this form, has a doctor prescribed any of the following pills or treatments?” Response categories included “pills for diabetes” and “insulin shots for diabetes.” The diabetes outcomes were ascertained, and the use of self-reported diabetes in the WHI has been validated and was consistent with medical records of documented treatment with anti-diabetes medications or for physician diagnosis of T2DM supported by laboratory measurements of glucose.^25,26^

### Computation of DII and E-DII Scores

Diet was evaluated with a standardized and validated self-administered food frequency questionnaire (FFQ) that was mailed to all participants at baseline.^27^ The FFQ was developed to estimate the average daily consumption of 122 food items over the previous 3-month period based on questions about the usual frequency of intake (from “never or less than once per month” to “2+ per day” for foods and “6+ per day” for beverages) and portion size (small, medium, or large compared with the stated medium portion size). Moreover, the WHI FFQ included information about the use of vitamin and mineral supplements as well as 19 adjustment questions about food preparation practices and types of added fats to permit more refined analyses of fat intake. The WHI FFQ was translated into Spanish, and 12 food items were added to reflect Hispanic eating patterns. The estimation of nutrient consumption of each participant was calculated using the University of Minnesota’s Nutrition Coordinating Center nutrient database.^28^

The procedure used to calculate the E-DII scores for all participants, from the FFQ, has been described elsewhere.^15,16^ Briefly, the DII was created after an extensive literature review of the pro- or anti-inflammatory effect of 45 dietary components or food parameters. Each food parameter received a parameter-specific overall inflammatory effect score that was calculated based on the pro-inflammatory, anti-inflammatory, or null effect of that dietary component in the scientific articles, taking into consideration the total number of articles published and the study design.^14,15^ Using the participants’ nutrients intake calculated from the FFQ, the *Z*-score and centered percentiles were calculated for each one of the available 32 food parameters for each study participant based on the energy-adjusted average and standard deviation from a global composite dataset created for this purpose.^15^ After this step, the centered proportion for each food parameter was multiplied by the respective overall food parameter-specific inflammatory effect score to obtain the food parameter-specific DII score. Finally, all the food parameter-specific DII scores were added together to create the overall DII score for an individual. The E-DII scores were computed similarly. However, all dietary intake was expressed per 1,000 kcal per day, and a global energy-adjusted comparative database was used to compute E-DII scores.^16^ DII/E-DII scores > 0 represent proinflammatory diets, and DII/E-DII scores ≤ 0 represent anti-inflammatory diets. The DII scores in the original study ranged from 7.98 (ie, strongly pro-inflammatory) to −8.87 (ie, strongly anti-inflammatory).^15^

In the WHI FFQ, 32 of the 45 original DII components were available for inclusion in the overall E-DII score (alcohol, vitamin B12, vitamin B6, *β*-carotene, caffeine, carbohydrate, cholesterol, energy, total fat, dietary fiber, folic acid, iron, magnesium, monounsaturated fatty acids, niacin, omega 3, omega 6, onion, protein, polyunsaturated fatty acids, riboflavin, saturated fat, selenium, thiamin, trans fat, vitamin A, vitamin C, vitamin D, vitamin E, zinc, green tea or black tea, and isoflavones). The components ginger, turmeric, garlic, oregano, pepper, rosemary, eugenol, saffron, flavan-3-ol, flavones, flavonols, flavonones, and anthocyanidins that were included in the original DII calculation were not included in the WHI FFQ or were not available in the nutrient database.^28,29^ Both the DII and E-DII scores were calculated without considering the use of nutritional supplements.

### Covariates

The study covariates were determined using baseline data from both the observational study and clinical trial components of the WHI study. Self-reported demographic information included age, ethnicity, and preferred language. Neighborhood socioeconomic status (NSES) was evaluated using a standardized geocoding protocol, which linked individual WHI participant addresses to the year 2000 US Census Federal Information Processing Standards codes and tract-level socioeconomic data. A summary measure of each participant’s neighborhood socioeconomic environment was estimated from the tract-level data using 6 variables representing several dimensions of wealth and income.^30^ Higher NSES scores represent a higher neighborhood socioeconomic status.

The following risk factors for T2DM were also examined: physical activity, smoking status, acculturation, alcohol intake, body mass index (BMI), and other chronic diseases. Physical activity was evaluated with a validated physical activity questionnaire, and it was included in the model as the total minutes of recreational physical activity per week, including walking and mild, moderate, and strenuous physical activity.^31^ Smoking status was determined as never or past or current smoker. Language preference (English or Spanish) was used as a proxy measure for acculturation status. Alcohol intake in grams per day was estimated using an FFQ.^27^ For BMI, weight was measured to the nearest 0.1 kg on a balance beam scale with light clothing and removal of shoes. Height was measured to the nearest 0.1 cm, using a wall-mounted Harpenden stadiometer. Body mass index was calculated as weight (kg) divided by the square of measured height (m^2^).^32^ Hypertension was defined as systolic pressure ≥130 mm Hg or diastolic ≥80 mm Hg or self-reported hypertension with the use of antihypertensive medication.^33^ Diagnosis of family history of T2DM at baseline was obtained from the medical history questionnaire in response to the question “Did your mother or father, or full-blooded sisters, full-blooded brothers, daughters, or sons ever have sugar diabetes or high blood sugar that first appeared as an adult?” Hypercholesterolemia was defined by self-report at baseline and then by use of lipid-modulating medication.^33^ Obesity was defined as a BMI ≥ 30. Diet quality was assessed using the Healthy Eating Index-2015 using the simple Healthy Eating Index scoring algorithm method.^34,35^

### Statistical Analysis

Two-sample *t*-tests were applied to compare the mean differences of each continuous variable between participants with and without diabetes. Chi-squared tests were used to compare the two groups on categorical variables. The DII/E-DII scores between Hispanic heritage groups were compared using one-way analysis of variance, and pairwise comparisons were made using the Tukey test. Separate Cox regression models were fit to examine the association between DII and E-DII scores at baseline and incident diabetes. The analyses were restricted only to follow-up events, and time-to-diabetes occurrence was the outcome of interest. Incident diabetes status was used as a dichotomous trait (0 = no, 1 = yes) as the indicator variable for failure/censorship. The survival time for participants who did not develop incident diabetes was defined as the days from enrollment to the end of follow-up (the follow-up time for diabetes events in this analysis includes data until September 2018). Hazard ratios (HR) and 95% confidence intervals (CI) are presented for each model. Cox regression models were conducted with and without adjusting for age at entry, lifestyle-related risk factors (smoking, alcohol intake, physical activity, and acculturation), known T2DM risk factors (family history of diabetes, hypertension, hypercholesterolemia, and BMI), and NSES. These covariates were serially added to the model, and the final model included all covariates. To determine whether the models with the DII or the E-DII scores would be used in the final analyses, the goodness of fit test using the *χ*^2^ statistic as well as the correlation between the DII/E-DII scores and diet quality was used. Multiple imputation with the fully conditional specification method was used to estimate missing values of the variable NSES (N = 237, which represents 7.5% of the observations) and physical activity (N = 139, which represents 4.5% of the observations), assuming that data were missing at random. Sensitivity analyses excluding women in the control arm of the dietary modification trial and considering the use of nutritional supplements in the calculation of the DII/E-DII scores were performed. The role of obesity as a potential effect modifier was evaluated by including an interaction between the DII/E-DII scores and obesity in the fully adjusted models. Analyses were performed using SAS software.^36^ All statistical tests were two-sided, and *P* ≤ 0.05 was considered statistically significant.

## RESULTS

**Figure 1.**
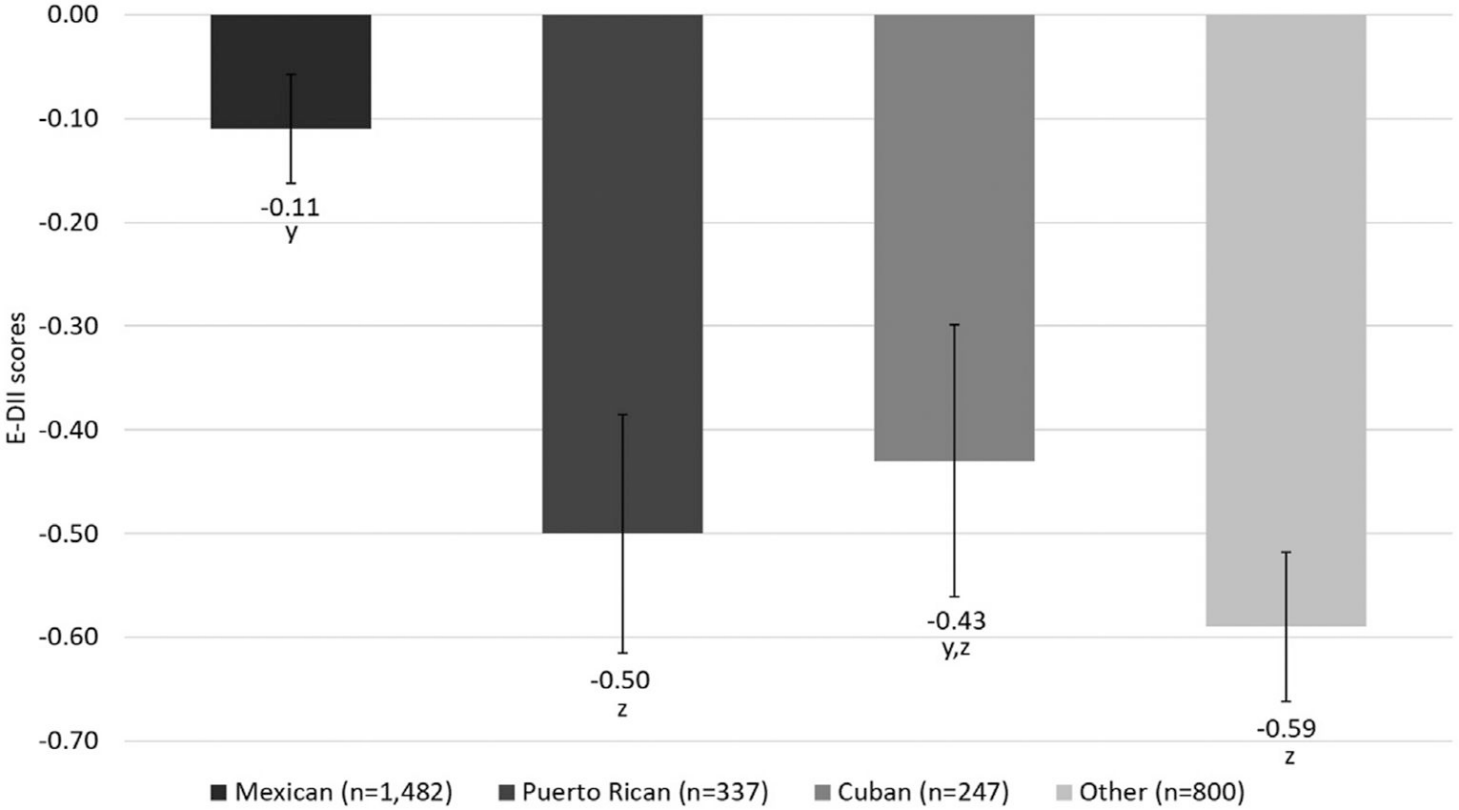
Average energy-adjusted dietary inflammatory index (E-DII) scores^a^ (±standard error) in the different Hispanic heritage groups^b,c^ of the Women’s Health Initiative. ^a^E-DII scores higher than zero represent pro-inflammatory diets with higher E-DII scores representing more inflammatory diets. E-DII scores lower than zero represent anti-inflammatory diets with lower E-DII scores representing more anti-inflammatory diets. ^b^Values not sharing a common superscript (y, z) are significantly different from each other using a Tukey test with *α* = 0.05. ^c^n = 2,866 due to missingness in the reporting of the Hispanic heritage groups.

Among 3,849 postmenopausal Hispanic women included in this study, 51.7% self-identified as having Mexican origin, 11.8% had Puerto Rican origin, 8.6% had Cuban origin, and 27.9% self-identified as other Hispanic/Latino ethnicity backgrounds. The incidence of diabetes was 13.1% during a median follow-up of 13.0 years (min: 0.3 years; max: 24.0 years). Women of Mexican and Puerto Rican ethnic descent had the highest incidence of diabetes (16.6%) followed by women in the category of other Hispanic/Latino ethnicity backgrounds (12.0%) and Cuban women (9.7%). The goodness-of-fit test showed that the E-DII better fit our data than the DII (E-DII *χ*^2^ = 224.0 and DII *χ*^2^ = 219.8), and the analysis of correlation with diet quality indicated that E-DII scores have a stronger correlation with diet quality than DII scores (E-DII *r* = −0.78 and DII *r* = −0.22), and therefore, the E-DII is used as the basis for all results presented. The mean E-DII score was −0.4 ± 2.1 (min: −6.7; max: 5.5). Women of Puerto Rican origin and women in the Other Hispanic/Latino ethnicity backgrounds category had higher anti-inflammatory scores than Mexican women (*P* < 0.05), and there was no statistically significant difference between the E-DII scores of Cuban women and all the other Hispanic heritage groups (*P* > 0.05) (Fig 1).

Women with incident diabetes compared with women without incident diabetes had higher E-DII scores (more pro-inflammatory diet scores) and reported higher intakes of total fat, saturated fat, trans fat, and cholesterol (*P* < 0.05) (Table 1). Women with incident diabetes compared with women without incident diabetes were also more likely to have a higher prevalence of hypertension, hypercholesterolemia, obesity, and a family history of diabetes, and to be in hormone treatment arm relative to their counterparts (*P* < 0.05). Women with incident diabetes also had lower age (the difference is not biologically significant), NSES, alcohol intake, and use of nutritional supplements, and they reported less time engaging in physical activity than women without incident diabetes (*P* < 0.05). Finally, no statistically significant differences were found in the prevalence of smoking, acculturation status (ie, preferred language), and intakes of energy, carbohydrates, dietary total sugar, protein, and dietary fiber between women with and without incident diabetes (*P* > 0.05).

Higher E-DII scores, representing more inflammatory diets, were associated with a higher risk of incident diabetes in fully adjusted models (HR, 1.09; 95% CI, 1.04–1.14); in other words, a 9% increase in T2DM risk per unit increase in E-DII score (Table 2). Sensitivity analyses showed that excluding participants from the control arm of the dietary modification trial and considering the use of nutritional supplements in the calculation of the E-DII scores did not change the HRs significantly (percentage change < 3%) (data not shown). Exploratory analysis that evaluated the association between DII scores (not adjusted by energy) and diabetes showed that the DII scores were not statistically significantly associated with the risk of diabetes (data not shown).

The interaction term between the E-DII scores and obesity was not statistically significant (*P* = 0.73) (Table 3). Exploratory analysis including an interaction term with BMI instead of obesity showed that the interaction term was not statistically significant either (*P* = 0.55).

## DISCUSSION

Higher E-DII scores were associated with a higher risk of incident diabetes among postmenopausal Hispanic women. Similar results were found in previous studies that evaluated the association between the DII/E-DII and T2DM or T2DM-related outcomes.^11,12,17,18^ The mechanism by which a proinflammatory diet can increase the risk of diabetes is proposed to include the modulation of the levels of inflammatory markers that increase insulin resistance, as well as serum lipids and glucose.^14,15,21,37^

In the current study, the non–energy-adjusted DII was not statistically significantly associated with the risk of incident diabetes. This may be because the adjustment by energy intake on the E-DII accounts for different eating patterns that are associated with positive and negative correlations between energy and nutrient intake, which also may explain the stronger correlation with diet quality of the E-DII in comparison with the DII.^16^

Women in the Other Hispanic/Latino ethnicity backgrounds category had the highest anti-inflammatory scores, followed by Puerto Rican women, Cuban women, and Mexican women. Similar results were found in the study conducted by Bermudez et. al. that evaluated the food intake of older Hispanic adults in the U.S. where Puerto Rican women had lower consumption of pro-inflammatory nutrients (protein, carbohydrates, and sugar) and higher consumption of anti-inflammatory nutrients (complex carbohydrates and unsaturated fats) than women in the “Other Hispanic” category.^38^ Different results were reported in the study conducted by Siega-Riz et. al. that evaluated the intake of nutrients in different Hispanic groups where results showed that participants of Mexican origin had a higher intake of anti-inflammatory nutrients (vitamin C, calcium, and fiber) than those of other Hispanic heritage groups; in comparison, participants of Cuban origin had the highest intake of pro-inflammatory nutrients (total energy, macronutrients, and alcohol) and participants of Puerto Rican origin had the lowest intake of anti-inflammatory nutrients (vitamin C, and fiber).^39^ The differences between the results may be explained by the differences in sex and age of the study populations because the study conducted by Siega-Riz et. al. included both men and women in an age range between 17-74 years in contrast with this study and the study conducted by Bermúdez et al^38^ that included older women.

Obesity did not modify the association between the E-DII and the risk of incident diabetes. The results suggest that increases in E-DII scores were associated with increased risk of incident diabetes of the same magnitude in women with and without obesity. This contrasts previous studies that found that the association between the DII and cardiovascular disease and interleukin-6 was stronger or only significant in participants with overweight or obesity but not in participants with normal weight.^22,23^ The differences in the results may suggest that obesity plays a role as an effect modifier of the association between dietary inflammation and cardiovascular disease but not in the association between dietary inflammation and T2DM. Another potential explanation for the inconsistencies in the results is the differences in the characteristics of the participants of the studies, because previous studies involved mostly non-Hispanic White participants, and one of the studies was conducted among university graduates so age, sex, and education level also could contribute to the observed differences.^23^

Adding lifestyle-related risk factors and known T2DM risk factors to the different models resulted in a modest attenuation of the HRs. This might be because these variables influence alternative pathways through which diet or diet quality can affect the risk of T2DM. Therefore, these variables may be considered mediators of the relationship between the E-DII and diabetes. Adding NSES to the models did not modify the HRs nor the statistical significance of the relationship between the E-DII and diabetes. This may be because socioeconomic status is also associated with T2DM risk factors, so, by simultaneously having T2DM risk factors and NSES in the model, we are controlling for the indirect effect of socioeconomic status over T2DM risk that is mediated by T2DM risk factors.^40^

Based on this study’s findings that pro-inflammatory diets are associated with the development of incident diabetes in Hispanic women, it is important to highlight the importance of addressing other factors associated with disparities in diet quality in Hispanic populations. Studies have shown that food insecurity is inversely associated with diet quality and therefore is associated with an increase in the risk of diet-related chronic disease in low-income adults.^41-43^ Rates of food insecurity are higher among Black and Hispanic adults than in non-Hispanic White adults.^41,44^ Moreover, many low-income and minority neighborhoods experience limited access to healthy foods (food deserts) or increased exposure to unhealthy foods (food swamps), which affects the quality of their diets.^45-47^ Neighborhood socioeconomic status has also been associated with diabetes risk in Hispanic women; higher NSES scores were associated with lower diabetes risk.^30^ Thus, future interventions to reduce dietary inflammation in Hispanic women should be accompanied by efforts to reduce food insecurity and improve food environments to reduce the risk of diabetes and other chronic diseases in this disadvantaged population.

This study has important implications for research on nutrition and health in older Hispanic populations. This is the first study to evaluate the association between the E-DII and the risk of T2DM in a diverse Hispanic population. Our findings provide a basis so that nutrition professionals can have a better understanding of the pathophysiological link between diet, inflammation, and T2DM in Hispanic women and use it to guide the development of lifestyle interventions to reduce the risk of T2DM in this population. Based on our findings, a diet with a lower consumption of pro-inflammatory nutrients (ie, carbohydrates, cholesterol, total fats, saturated fats, trans fats, protein, Fe, and vitamin B12) and a higher consumption of anti-inflammatory nutrients (ie, dietary fiber, folic acid, unsaturated fats, and vitamins A, C, D, and E), is consistent with the Dietary Guidelines for Americans and can help to reduce the risk of T2DM.^48^ Moreover, this study adds to the literature regarding the link between the E-DII and chronic noncommunicable diseases. Our findings show that the E-DII provides a noninvasive tool to assess the inflammatory potential of the diet, and future research can assess whether dietary interventions have an effect on reducing people’s E-DII scores. Lifestyle interventions that promote healthy diets while adapting dietary recommendations to the cultural preferences of Hispanic women are needed to help reduce health disparities in this population.

The strengths of this study include the large Hispanic study population from diverse heritage backgrounds and a prospective longitudinal study design. Additional strengths include the validation of self-reported diabetes in the WHI through concordance of medical records, documented treatment of antidiabetic medications, or physician diagnosis of T2DM supported by laboratory measurements of glucose.^25,26^ Another strength is that the WHI FFQ was designed to reflect the diverse, regional, and ethnic eating patterns of the United States, including Hispanic eating patterns. In addition, many physical, biological, and social covariates were available in the WHI database to reduce the residual confounding of the models. Finally, the DII has been validated to predict concentrations of inflammatory markers in the WHI.^29^

Limitations of this study include the use of only baseline dietary information that did not allow for evaluation of changes in the inflammatory potential of diet over time and measurement errors in the dietary assessment that is associated with self-report of diet.^49,50^ In addition, there was a short recall period in the WHI FFQ (only 3 months) and 13 missing anti-inflammatory components from the original DII calculation. However, in the initial validation of the DII, the loss of even more parameters did not reduce the ability to predict interval changes in C-reactive protein values over a year.^51^

## CONCLUSIONS

Pro-inflammatory diets, as measured by higher E-DII scores, were associated with a higher risk of incident diabetes among postmenopausal Hispanic women. Obesity was not shown to modify the effect of the E-DII over the risk of incident diabetes. Future research is needed to understand how diets’ inflammatory potential can be decreased, especially across diverse ethnicities, to reduce the impact of chronic diseases, including diabetes.

## Figures and Tables

**Table 1.** Descriptive characteristics by diabetes status of Hispanic women from the Women’s Health Initiative (n = 3,849)

	Incident Diabetes (n = 505)	No-Incident Diabetes (n = 3,344)	
TVariable	Mean ± SD	Mean ± SD	*P* ^a^
Age (y)	59.6 ± 6.4	60.2 ± 6.9	0.04
E-DII^b^ (score)	0.1 ± 2.0	−0.4 ± 2.0	<0.0001
Neighborhood SES^c^ (score)	66.7 ± 10.7	69.3 ± 10.2	<0.0001
Physical activity (h/wk)	141.2 ± 175.9	171.4 ± 186.9	0.001
Alcohol intake (g/d)	0.9 ± 5.1	1.4 ± 3.5	0.007
BMI^d^	31.0 ± 5.5	28.0 ± 5.4	<0.0001
Energy intake (kcal/d)	1,661 ± 793	1,603 ± 760	0.11
Carbohydrates (g/d)	205.3 ± 96.6	202.9 ± 94.5	0.60
Total fat (g/d)	64.6 ± 38.9	59.2 ± 35.7	0.002
Saturated fat (g/d)	21.0 ± 13.1	19.4 ± 12.5	0.007
Trans fat (g/d)	4.1 ± 3.0	3.7 ± 2.8	0.001
Cholesterol (mg/d)	243.3 ± 154.5	222.4 ± 148.9	0.004
Protein (g/d)	66.9 ± 34.0	65.6 ± 33.9	0.44
Total dietary sugar (g/d)	94.5 ± 57.6	94.5 ± 53.5	0.99
Dietary fiber (g/d)	15.2 ± 7.7	15.4 ± 7.8	0.65
Variable	n (%)	n (%)	*P* ^e^
Pro-inflammatory diet (yes)	274 (54.3)	1,448 (43.4)	<0.0001
Obesity (yes)	267 (53.6)	944 (28.5)	<0.0001
Smoking (yes)	193 (39.1)	1,183 (36.1)	0.20
Hypertension (yes)	326 (64.6)	1,791 (53.6)	<0.0001
Hypercholesterolemia (yes)	87 (19.1)	427 (13.8)	0.003
Family history of diabetes (yes)	274 (54.8)	1,265 (38.5)	<0.0001
Nutritional supplements use (yes)	239 (47.3)	1,797 (53.7)	0.007
Preferred language (English)	373 (73.9)	2,562 (76.6)	0.18
Hormone treatment arm (yes)	166 (32.9)	719 (21.5)	<0.0001

a *P*-values are for two-sample *t*-tests comparing characteristics of Hispanic women with and without incident diabetes.

b E-DII = energy-adjusted dietary inflammatory index.

c SES = socioeconomic status.

d BMI = body mass index.

e *P*-values are for *χ*^2^ tests comparing characteristics of Hispanic women with and without incident diabetes.

**Table 2.** Association of energy-adjusted dietary inflammatory index (E-DII) scores and incident diabetes in Hispanic women from the Women’s Health Initiative (n = 3,751)^a^

Model	Variables	Hazard Ratio	95% CI	*P* ^b^
1^c^	Model 1	1.13	1.08, 1.18	<0.0001
2^d^	Model 1 + lifestyle-related risk factors	1.11	1.06, 1.16	<0.0001
3^e^	Model 2 + known T2DM^f^ risk factors	1.09	1.04, 1.14	0.0004
4^g^	Model 3 + NSES^h^	1.09	1.04, 1.14	0.0004

a The sample size does not equal 3,849 because of the missingness of responses in some covariates.

b *P*-values are for Cox regression models.

c Model 1: adjusted by age at baseline.

d Model 2: adjusted by age at baseline, and lifestyle-related risk factors.

e Model 3: adjusted by age at baseline, lifestyle-related risk factors, and known T2DM risk factors.

f T2DM: Type 2 diabetes mellitus.

g Model 4: adjusted by age at baseline, lifestyle-related risk factors, known T2DM risk factors, and NSES.

h NSES: neighborhood socioeconomic status.

**Table 3.** Test for interaction between the energy-adjusted dietary inflammatory index scores and obesity in Hispanic women from the Women’s Health Initiative (n = 3,751)^a^

Models	*P*-Value of interaction term^b^
Model 4^c^ + E-DII^d^*obesity	0.73
Model 4 + E-DII*BMI^e^	0.55

a The sample size does not equal 3,849 because of the missingness of responses in some covariates.

b *P*-values are for interaction terms in Cox regression models.

c Model 4 is adjusted by age at baseline, lifestyle-related risk factors, known type 2 diabetes risk factors, and neighborhood socioeconomic status.

d E-DII = energy-adjusted dietary inflammatory index.

e BMI = body mass index.

## RESEARCH SNAPSHOT

### Research Question:

Are the dietary inflammatory index (DII) and energy-adjusted DII (E-DII) associated with incident diabetes in self-identified Hispanic women from the Women’s Health Initiative? Does obesity modify the association between the DII scores and incident diabetes?

### Key Findings:

Results showed that pro-inflammatory diets were associated with a higher risk of diabetes and that obesity did not modify the effect of the energy-adjusted dietary inflammatory index (E-DII) on the risk of diabetes.

## References

[1] Centers for Disease Control. Long-Term Trends in Diabetes. 2017. Accessed February 19, 2023. http://www.cdc.gov/diabetes/data

[2] World Health Organization. Diabetes. 2022. Accessed February 19, 2023. https://www.who.int/news-room/fact-sheets/detail/diabetes

[3] Spanakis EK, Golden SH. Race/ethnic difference in diabetes and diabetic complications. Curr Diab Rep. 2013;13(6):814–823. 24037313 10.1007/s11892-013-0421-9 PMC3830901

[4] Centers for Disease Control. National Diabetes Statistics Report. 2022. Accessed February 19, 2023. https://www.cdc.gov/diabetes/data/statistics-report/index.html?ACSTrackingID=DM72996&ACSTrackingLabel=New%20Report%20Shares%20Latest%20Diabetes%20Stats%20&deliveryName=DM72996

[5] CDC. National Diabetes Statistics Report 2020. Estimates of diabetes and its burden in the United States. Published online 2020. Accessed July 31, 2023. https://www.cdc.gov/diabetes/pdfs/data/statistics/national-diabetes-statistics-report.pdf

[6] Coleman-Jensen A, Nord M, Singh A. Household food security in the United States in 2012. Washington, DC: US Department of Agriculture, Economic Research Service; 2013. Report No. ERR–155.

[7] Mainous AG, Diaz VA, Geesey ME. Acculturation and healthy lifestyle among Latinos with diabetes. Ann Fam Med. 2008;6(2):131–137. 18332405 10.1370/afm.814 PMC2267424

[8] Santiago-Torres M, Tinker LF, Allison MA, Development and use of a traditional Mexican diet score in relation to systemic inflammation and insulin resistance among women of Mexican descent. J Nutr. 2015;145(12):2732–2740. 26491126 10.3945/jn.115.213538 PMC4656903

[9] Hui Q, Asadi A, Park YJ, Amyloid formation disrupts the balance between interleukin-1*β* and interleukin-1 receptor antagonist in human islets. Mol Metab. 2017;6(8):833–844. 28752047 10.1016/j.molmet.2017.05.016 PMC5518725

[10] Wieser V, Moschen AR, Tilg H. Inflammation, cytokines and insulin resistance: A clinical perspective. Arch Immunol Ther Exp (Warsz). 2013;61(2):119–125. 23307037 10.1007/s00005-012-0210-1

[11] Vahid F, Shivappa N, Karamati M, Naeini AJ, Hebert JR, Davoodi SH. Association between dietary inflammatory index (DII) and risk of prediabetes: A case-control study. Appl Physiol Nutr Metabol. 2017;42(4):399–404. 10.1139/apnm-2016-0395 28177734

[12] van Woudenbergh GJ, Theofylaktopoulou D, Kuijsten A, Adapted dietary inflammatory index and its association with a summary score for low-grade inflammation and markers of glucose metabolism: The Cohort study on Diabetes and Atherosclerosis Maastricht (CODAM) and the Hoorn study. Am J Clin Nutr. 2013;98(6):1533–1542. 24153342 10.3945/ajcn.112.056333

[13] Calle MC, Fernandez ML. Inflammation and type 2 diabetes. Diabetes Metab. 2012;38(3):183–191. 22252015 10.1016/j.diabet.2011.11.006

[14] Cavicchia PP, Steck SE, Hurley TG, A new dietary inflammatory index predicts interval changes in serum high-sensitivity C-reactive protein. J Nutr. 2009;139(12):2365–2372. 19864399 10.3945/jn.109.114025 PMC2777480

[15] Shivappa N, Steck SE, Hurley TG, Hussey JR, Hébert JR. Designing and developing a literature-derived, population-based Dietary Inflammatory Index. Public Health Nutr. 2014;17(8):1689–1696. 23941862 10.1017/S1368980013002115 PMC3925198

[16] Hébert JR, Shivappa N, Wirth MD, Hussey JR, Hurley TG. Perspective: The dietary inflammatory index (DII): Lessons learned, improvements made, and future directions. Adv Nutr. 2019;10(2):185–195. 30615051 10.1093/advances/nmy071 PMC6416047

[17] Denova-Gutiérrez E, Muñoz-Aguirre P, Shivappa N, Dietary inflammatory index and type 2 diabetes mellitus in adults: The diabetes mellitus survey of mexico city. Nutrients. 2018;10(4):385. 29561774 10.3390/nu10040385 PMC5946170

[18] Mtintsilana A, Micklesfield LK, Chorell E, Adiposity mediates the association between the dietary inflammatory index and markers of type 2 diabetes risk in middle-aged black South African women. Nutrients. 2019;11(6):1246. 31159253 10.3390/nu11061246 PMC6628082

[19] Levin KA. Study design V: Case–control studies. Evid Based Dent. 2006;7(3):83–84. 17003803 10.1038/sj.ebd.6400436

[20] Levin KA. Study design III: Cross-sectional studies. Evid Based Dent. 2006;7(1):24–25. 16557257 10.1038/sj.ebd.6400375

[21] Hariharan R, Odjidja EN, Scott D, The dietary inflammatory index, obesity, type 2 diabetes, and cardiovascular risk factors and diseases. Obes Rev. 2022;23(1):e13349. 34708499 10.1111/obr.13349

[22] Tabung FK, Steck SE, Ma Y, The association between dietary inflammatory index and risk of colorectal cancer among postmenopausal women: Results from the Women’s Health Initiative. Cancer Causes & Control. 2015;26(3):399–408. 25549833 10.1007/s10552-014-0515-y PMC4334706

[23] Ramallal R, Toledo E, Martínez-González MA, Dietary inflammatory index and incidence of cardiovascular disease in the SUN cohort. PLoS One. 2015;10(9):e0135221. 26340022 10.1371/journal.pone.0135221 PMC4560420

[24] Design of the Women’s Health Initiative Clinical Trial and Observational Study. Control Clin Trials. 1998;19(1):61–109. 9492970 10.1016/s0197-2456(97)00078-0

[25] Margolis KL, Lihong Qi, Brzyski R, Validity of diabetes self-reports in the Women’s Health Initiative: Comparison with medication inventories and fasting glucose measurements. Clinical Trials. 2008;5(3):240–247. 18559413 10.1177/1740774508091749 PMC2757268

[26] Jackson JM, DeFor TA, Crain AL, Validity of diabetes self-reports in the Women’s Health Initiative. Menopause. 2014;21(8):861–868. 24496083 10.1097/GME.0000000000000189 PMC4278641

[27] Patterson RE, Kristal AR, Tinker LF, Carter RA, Bolton MP, Agurs-Collins T. Measurement characteristics of the Women’s Health Initiative food frequency questionnaire. Ann Epidemiol. 1999;9(3):178–187. 10192650 10.1016/s1047-2797(98)00055-6

[28] NDSR Software Food and Nutrient Database [Computer software]. Version 2013. Minneapolis, MN: Nutrition Coordinating Center (NCC), University of Minnesota; 2013.

[29] Tabung FK, Steck SE, Zhang J, Construct validation of the dietary inflammatory index among postmenopausal women. Ann Epidemiol. 2015;25(6):398–405. 25900255 10.1016/j.annepidem.2015.03.009 PMC4433562

[30] Qi L, Nassir R, Kosoy R, Relationship between diabetes risk and admixture in postmenopausal African-American and Hispanic-American women. Diabetologia. 2012;55(5):1329–1337. 22322919 10.1007/s00125-012-2486-4 PMC4430092

[31] Johnson-Kozlow M, Rock CL, Gilpin EA, Hollenbach KA, Pierce JP. Validation of the WHI brief physical activity questionnaire among women diagnosed with breast cancer. Am J Health Behav. 2007;31(2):193–202. 17269909 10.5555/ajhb.2007.31.2.193

[32] Garrow JS, Webster J. Quetelet’s index (W/H2) as a measure of fatness. Int J Obes. 1985;9(2):147–153. 4030199

[33] Hsia J, Margolis KL, Eaton CB, Prehypertension and cardiovascular disease risk in the Women’s Health Initiative. Circulation. 2007;115(7):855–860. 17309936 10.1161/CIRCULATIONAHA.106.656850

[34] Krebs-Smith SM, Pannucci TE, Subar AF, Update of the Healthy Eating Index: HEI-2015. J Acad Nutr Diet. 2018;118(9):1591–1602. 30146071 10.1016/j.jand.2018.05.021 PMC6719291

[35] National Cancer Institute. Choosing a Method; 2022. Accessed February 19, 2023. https://epi.grants.cancer.gov/hei/tools.html. 2022

[36] [Computer software]. Version 9.4. Cary, NC: SAS Institute Inc; 2013.

[37] Giugliano D, Ceriello A, Esposito K. The effects of diet on inflammation. J Am Coll Cardiol. 2006;48(4):677–685. 16904534 10.1016/j.jacc.2006.03.052

[38] Bermúdez OI, Falcón LM, Tucker KL. Intake and food sources of macronutrients among older Hispanic adults: Association with ethnicity, acculturation, and length of residence in the United States. J Am Diet Assoc. 2000;100(6):665–673. 10863569 10.1016/s0002-8223(00)00195-4

[39] Siega-Riz AM, Sotres-Alvarez D, Ayala GX, Food-group and nutrient-density intakes by Hispanic and Latino backgrounds in the Hispanic Community Health Study/Study of Latinos. Am J Clin Nutr. 2014;99(6):1487–1498. 24760972 10.3945/ajcn.113.082685 PMC4021787

[40] Volaco A, Cavalcanti AM, Filho RP, Precoma DB. Socioeconomic status: The missing link between obesity and diabetes mellitus? Curr Diabetes Rev. 2018;14(4):321–326. 28637406 10.2174/1573399813666170621123227

[41] Hernandez DC, Reesor LM, Murillo R. Food insecurity and adult overweight/obesity: Gender and race/ethnic disparities. Appetite. 2017;117:373–378. 28739148 10.1016/j.appet.2017.07.010

[42] Brown AGM, Esposito LE, Fisher RA, Nicastro HL, Tabor DC, Walker JR. Food insecurity and obesity: Research gaps, opportunities, and challenges. Transl Behav Med. 2019;9(5):980. 31570918 10.1093/tbm/ibz117 PMC6937550

[43] Leung CW, Epel ES, Ritchie LD, Crawford PB, Laraia BA. Food insecurity is inversely associated with diet quality of lower-income adults. J Acad Nutr Diet. 2014;114(12):1943–1953.e2. 25091796 10.1016/j.jand.2014.06.353

[44] Coleman-Jensen A, Rabbitt MP, Gregory CA, Singh A. Statistical supplement to household food security in the United States in 2017. 2018. Accessed December 15, 2021. www.ers.usda.gov

[45] Walker RE, Keane CR, Burke JG. Disparities and access to healthy food in the United States: A review of food deserts literature. Health Place. 2010;16(5):876–884. 20462784 10.1016/j.healthplace.2010.04.013

[46] Stowers KC, Jiang Q, Atoloye A, Lucan S, Gans K. Racial differences in perceived food swamp and food desert exposure and disparities in self-reported dietary habits. Int J Environ Res Public Health. 2020;17(19):1–14. 10.3390/ijerph17197143 PMC7579470 33003573

[47] Lucan SC, Maroko AR, Patel AN, Gjonbalaj I, Elbel B, Schechter CB. Healthful and less-healthful foods and drinks from storefront and non-storefront businesses: implications for “food deserts,” “food swamps” and food-source disparities. Public Health Nutr. 2020;23(8):1428–1439. 32223780 10.1017/S1368980019004427 PMC7196006

[48] Dietary Guidelines for Americans. Dietary Guidelines for Americans, 2020-2025 and Online Materials. 2020. Accessed February 19, 2023. https://www.dietaryguidelines.gov/resources/2020-2025-dietary-guidelines-online-materials

[49] Schoeller DA. Limitations in the assessment of dietary energy intake by self-report. Metabolism. 1995;44(2 Suppl 2):18–22. 10.1016/0026-0495(95)90204-x 7869932

[50] Orchard T, Yildiz V, Steck SE, Dietary inflammatory index, bone mineral density, and risk of fracture in postmenopausal women: Results from the Women’s Health Initiative. J Bone Miner Res. 2017;32(5):1136–1146. 28019686 10.1002/jbmr.3070 PMC5413384

[51] Shivappa N, Steck SE, Hurley TG, A population-based dietary inflammatory index predicts levels of C-reactive protein in the Seasonal Variation of Blood Cholesterol Study (SEASONS). Public Health Nutr. 2014;17(8):1825–1833. 24107546 10.1017/S1368980013002565 PMC3983179

